# Relationship between Blood Lipid Profiles and Risk of Lupus Nephritis in Children

**DOI:** 10.1155/2022/6130774

**Published:** 2022-10-26

**Authors:** Jiajia Liu, Wenqi Song, Dawei Cui

**Affiliations:** ^1^Department of Clinical Laboratory Center, Beijing Children's Hospital, Capital Medical University, National Center for Children's Health, Beijing, China; ^2^The First Affiliated Hospital, Zhejiang University School of Medicine, Hangzhou, China

## Abstract

**Objective:**

Systemic lupus erythematosus (SLE) is a relatively common rheumatic disease in children. The characteristics of blood lipid metabolism in children with LN are little reported. This study aimed to explore the relationship between blood lipid profiles and the risk of lupus nephritis (LN) in children.

**Methods:**

A total of 134 children with newly diagnosed SLE were divided into LN and non-LN groups according to pathological renal biopsy results. Clinical manifestations and blood lipid profiles were analyzed and compared between the two groups, and the relationships between blood lipid profiles and risk of LN were evaluated.

**Results:**

The positivity rate of an anti-dsDNA antibody and an SLE disease activity index (SLEDAI) were significantly increased, and C3 and C4 levels were significantly reduced in the LN compared with the non-LN group. The overall incidence of dyslipidemia was 79.9%, with a significantly high incidence in the LN group compared with the non-LN group. Total cholesterol (TC), triglycerides (TG), low-density lipoprotein cholesterol (LDL-C), and very LDLC (VLDL-C) were all higher in the LN group than those in the non-LN group. However, there was no significant difference in high-density lipoprotein cholesterol (HDL-C) between the two groups. The blood lipid levels were positively correlated with 24-hour urine protein quantification, urea, creatinine, uric acid, urinary IgG, urinary microalbumin, urinary transferrin, urinary *α*1 microglobulin, and urinary N-acetyl glucosidase, respectively. Receiver-operating characteristic curves showed that combined detection of TC, TG, LDL-C, and VLDL-C had higher discrimination capacity than that in individual measures. Additionally, increased TC was independently associated with the occurrence of LN.

**Conclusions:**

Children with LN have significant dyslipidemia. High levels of TC, TG, LDL-C, and VLDL-C are closely related to the occurrence of pLN. Clinical attention should be paid to monitoring and managing blood lipid profiles in children with LN.

## 1. Introduction

Systemic lupus erythematosus (SLE) is a chronic multisystem autoimmune disease resulting in multiple organ damage [1]. SLE is a relatively common rheumatic disease in children, referred to as pediatric SLE (pSLE). Compared with adult patients, children have a more active disease course and more serious end-organ damage [2, 3]. Lupus nephritis (LN) is one of the most common complications of SLE, especially pSLE, with up to 70% of children with pSLE developing LN. Childhood LN is often more serious and progresses more rapidly to organ damage and has higher disease activity compared with adult-onset LN, resulting in higher mortality rates of pSLE versus adult-onset SLE [3, 4].

A variety of substance metabolism disorders occur in patients with SLE, mediated by the immune response and inflammation. Dyslipidemia is believed to decisively influence the long-term prognosis of SLE patients, affecting both cardiovascular events and LN [3]. Previous studies mostly focused on the relationship between blood lipid levels and the progression of cardiovascular disease in SLE. One clinical study showed that the risks of myocardial infarction and cardiovascular disease mortality were significantly higher in SLE patients with LN than those without LN [5]. However, few studies have examined the correlation between lipid metabolism and the occurrence of LN, especially in pSLE.

The current study aimed to explore the characteristics of blood lipid metabolism in children with LN and to estimate the association between relatively complete lipid profiles and the risk of LN, to provide a valuable reference for the clinical management of blood lipids and the prevention of LN in children.

## 2. Materials and Methods

### 2.1. Study Participants

This study was conducted at the Division of Rheumatology, Beijing Children's Hospital, Capital Medical University, China, from January 2020 to December 2021, with approval from the Beijing Children's Hospital research ethics committee. This retrospective longitudinal study included 134 patients with newly diagnosed SLE, who were aged <18 years at the time of diagnosis. All the patients met the American College of Rheumatology/European League against Rheumatism revised 2019 criteria for SLE. Sixty-five patients had renal biopsy-proven LN (LN group), and 69 had negative urinalysis results (urinary protein excretion <0.5 g/day or less than three and without casts, e.g., red cells, granular, or mixed casts) and normal renal function (non-LN group).

The exclusion criteria were as follows: age >18 years; diagnosis of Sjögren's syndrome, mixed connective tissue disease, or other autoimmune diseases; diagnosis of chronic kidney disease (CKD), diabetes mellitus, essential hypertension, liver or thyroid disease, or familial hyperlipidemia; and children receiving lipid-lowering therapy.

Another 70 children who underwent a physical examination during the same period were selected as the healthy control group.

The study was conducted in accordance with the principles of the Declaration of Helsinki, 1975. Signed informed consent was obtained from the children's parents and/or legal guardians.

### 2.2. Clinical Data Collection and Laboratory Assessment

Baseline data, including sex, age, body mass index (BMI), clinical symptoms, were recorded retrospectively. Within two to five days of admission, before trying any treatment, cubital venous blood and urine specimens were collected from each patient for biochemical and immune parameters, and then, laboratory data were recorded and analyzed: blood lipid profiles including total cholesterol (TC), triglycerides (TGs), high-density lipoprotein cholesterol (HDL-C), low-density lipoprotein cholesterol (LDL-C), and very LDL-C (VLDL-C), nonesterified fatty acid; fasting blood glucose (FBG); erythrocyte sedimentation rate (ESR); complement C3 and C4; specific antibodies including anti-double-stranded DNA (anti-dsDNA) and anti-Smith (anti-Sm); 24 h urine protein quantification (24 h M-TP); kidney function indicators including urea, creatinine, and uric acid; early renal injury markers including urinary IgG, urinary microalbumin (mAlb), urinary transferrin (TRU), and urinary *α*1 microglobulin (A1M); and N-acetyl glucosidase (NAG).

SLE severity was assessed at the enrollment visit using the SLE disease activity index (SLEDAI) as follows: (1) no activity (score: 0–4), (2) mild-to-moderate activity (score: 5–14), and (3) high activity (score: ≥15).

### 2.3. Statistical Analysis

The data were analyzed, and images were created using SPSS 24.0 (SPSS Inc., Chicago, IL, USA). Quantitative data were expressed as a mean ± standard deviation, and categorical variables, such as sex, SLE disease activity, and dyslipidemia, were presented as percentages. Differences between categorical variables were determined by *χ*^2^ tests, and differences between continuous variables were determined using the *t*-tests for independent samples and Wilcoxon's rank-sum test. Correlations between parametric variables were evaluated using Pearson's correlation curve. Receiver-operating characteristic (ROC) curves were drawn for LN predictor variables, and optimal cutoffs were confirmed by maximizing the sum of sensitivity and specificity. The coefficient of univariate logistic regression and the odds ratio (OR), including the 95% confidence interval (CI), were calculated to identify variables associated with the presence of LN. Predictors that were significantly correlated with the presence of LN in univariate logistic regression analysis were analyzed by stepwise regression in multivariate analysis, to identify independent risk factors for LN. *P* < 0.05 was considered significant.

## 3. Results

### 3.1. Demographic and Disease-Related Data

We enrolled 134 children with pSLE, including 65 in the LN group (13 males, 52 females, age 5–17 (10.23 ± 2.57) years, and BMI (21.96 ± 7.91) kg/m^2^) and 69 in the non-LN group (17 males, 52 females, age 5–17 (10.64 ± 2.53) years, and BMI (20.73 ± 2.03) kg/m^2^). There were no significant differences in sex, age, or BMI between the two groups (*P* > 0.05 for all analyses). There were also no significant differences in FBG, ESR, or anti-Sm antibody positivity rate in peripheral blood between the LN and non-LN groups (all *P* > 0.05). However, C3/C4 levels were significantly lower (both *P* < 0.05), and the anti-dsDNA antibody positivity rate was significantly higher (76.9% vs. 59.4%, *P* < 0.05) in the LN group than in the non-LN group. The SLEDAI scores were also significantly higher in the LN group than in the non-LN group (*P* < 0.05). The overall incidence of dyslipidemia was 79.9%, and the incidence was significantly higher in the LN than in the non-LN group (89.2% vs. 71.0%, *P* < 0.05) (Table 1).

### 3.2. Comparison of Blood Lipid Profiles among Groups

Regarding blood lipid profiles, children with SLE had higher TG, LDL-C, and VLDL-C levels and lower HDL-C levels than healthy children in both the LN and non-LN groups, indicating that children with SLE had disordered lipid profiles. There was no significant difference in TC between the non-LN group and healthy children, but levels of TC, TG, LDL-C, and VLDL-C were all significantly higher in the LN group than in the non-LN group (*P* < 0.05 for all analyses). However, there was no significant difference in HDL-C levels between the LN and the non-LN groups (*P* > 0.05) (Table 2).

### 3.3. ROC Curve Analysis for Blood Lipid Profiles and Predictive Values for LN in Children

**Figure 1:**
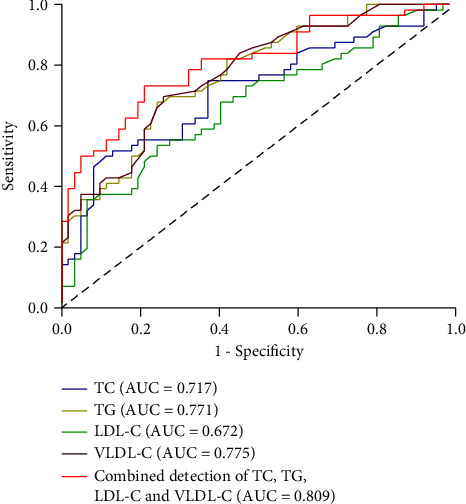
ROC curve analysis of the use of blood lipid profiles for diagnosis of LN. Abbreviations: TC: total cholesterol; TG: triglyceride; LDL-C: low-density lipoprotein cholesterol; VLDL-C: very low-density lipoprotein cholesterol.

We determined the abilities of TC, TG, LDL-C, and VLDL-C to distinguish between children with and without LN using ROC curves. The respective ROC cutoff values were 5.23, 1.64, 3.52, and 0.32 mmol/L, respectively (Figure 1), and the respective area under the curve (AUC) values were 0.717, 0.771, 0.672, and 0.775. Based on these results, the combined detection of TC, TG, LDL-C, and VLDL-C had higher capacity for discrimination than any individual measures, with an AUC of 0.809 (95% CI: 0.731–0.887, *P* < 0.05) (Figure 1).

### 3.4. Associations between Blood Lipid Profiles and Renal Function Indicators in Children with LN

Pearson's correlation analysis revealed significant positive correlations between TC, TG, LDL-C, and VLDL-C and kidney function indicators (Table 3) but not between HDL-C levels and kidney function indicators (all *P* > 0.05). Thus, these results suggest that TC, TG, LDL-C, and VLDL-C may be useful predictors of disease severity in children with LN. In addition, we analyzed the correlation between blood lipid profiles and immunological indicators, such as complement C3, C4, anti-dsDNA, and anti-Sm, and we found no significant difference (relevant data not shown in this article).

### 3.5. Multivariable Analysis of Relationships of Lipid Profiles and Other Parameters with LN

**Figure 2:**
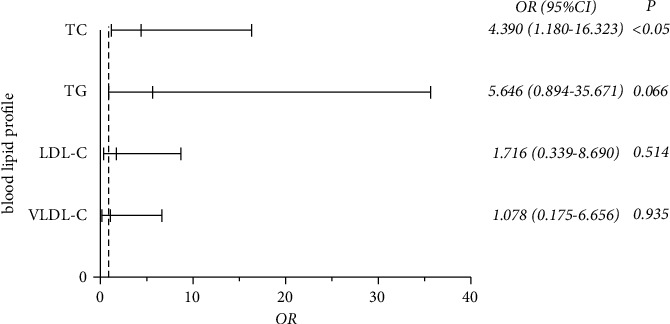
Multivariate logistic regression analysis between blood lipid profiles and LN Abbreviations: TC: total cholesterol; TG: triglyceride; LDL-C: low-density lipoprotein cholesterol; VLDL-C: very low-density lipoprotein cholesterol.

The ORs and 95% CIs of the risk factors of pLN are shown in Figure 2. On the basis of the previous results, we determined that high TC levels (≥5.23 mmol/L) increased the risk of LN in children in a logistic regression model after adjusting other variables such as BMI, age, and sex (OR = 4.390; 95% CI = 1.180–16.323; *P* < 0.05). These findings demonstrated that TC was independently associated with the occurrence of LN and may thus be a better risk predictor for LN.

## 4. Discussion

SLE is a chronic inflammatory autoimmune disease that affects many organs, including the skin, joints, lungs, heart, kidneys, and nervous system. LN is the most common type of organ damage in patients with SLE, occurring in up to 40% of patients [1, 5, 6]. The incidence rate of LN in children is significantly higher than that in adults, with a younger onset age of LN associated with a worse prognosis. Artim-Esen et al. [7] reported a higher frequency in the pediatric SLE (pSLE) group of renal involvement than in adult-onset SLE (aSLE). A significant proportion of patients in the pSLE group had damage, most prominently in the renal domain. These children were described as having a more severe disease course, with higher disease activity and worse prognosis. After investigation, Torrente-Segarra et al. [8] also confirmed that pSLE patients more frequently underwent dialysis and kidney transplantations. The timely detection of renal involvement and its appropriate treatment are key to preventing renal damage [9, 10].

Some studies have found significant changes in lipid metabolism in SLE patients, including the “lupus model” of dyslipoproteinemia [11], abnormal chylomicron metabolism [12], and enhanced lipid peroxidation [13]. Dyslipidemia is closely related to inflammation, autoimmunity, and oxidative stress in SLE patients [13, 14]. An examination of blood lipids showed that children with SLE had obvious lipid metabolism disorders in the early stage, and they could be divided into the following types: increased serum TC level, increased serum TG level, increased serum LDL level, and decreased serum HDL level [15]. Some cross-sectional analytical studies found dyslipidemia rates of about 50%–85% in children with SLE [16, 17]; however, no epidemiological studies have examined the blood lipid levels in children with LN. The incidence of dyslipidemia in children with LN in the current study was 89.2%, which was significantly higher than that in children without LN. We also showed that the main forms of dyslipidemia in the LN group, compared with children without LN, had significant increases in TC, TG, LDL, and VLDL. The timely monitoring of blood lipids in children with SLE will thus play an important role in improving the prognosis of the disease.

The specific pathogenesis of LN is still unclear. Some researchers have proposed the “lipid nephrotoxicity hypothesis,” which states that lipid metabolism disorders can promote and/or aggravate glomerular and tubulointerstitial lesions by changing the lipid homeostasis of renal tissue. In addition to the deposition of immune complexes, the kidneys may also have abnormal lipid deposition in patients with LN, leading to glomerular capillary endothelial cell damage. Infiltrated monocytes and macrophages phagocytose lipids and are then transformed into foam cells [18]. Thus, hyperlipidemia may play a vital role in the development of glomerular atherosclerosis in renal disease. Some studies found that hyperlipidemia caused damage to the glomerular interstitium. In SLE patients with hyperlipidemia, lipids are deposited on the glomerular wall and then embedded in the glomerular lumen, where they are encapsulated by foam cells, leading to blockage of the entire lumen. In contrast, patients without hyperlipidemia show no such damage to the glomerular interstitium. Dyslipidemia may cause renal damage in SLE patients through the above-mentioned mechanisms, and abnormal blood lipids may sometimes be the first manifestation of LN. Chong et al. [19] compared the lipid profiles of 100 Chinese patients with quiescent LN with 100 controls with nonlupus, nondiabetic CKD, matched for sex, age, and renal function. They found that dyslipidemia was prevalent in LN patients and was more severe than in controls with a similar degree of CKD despite disease quiescence, a low steroid dose, and a low level of proteinuria. McKinley et al. [20] found that hyperlipidemia was an important risk factor associated with higher mortality and the development of renal failure in patients with LN. A cohort study of patients with SLE at the University of Toronto also showed that elevated serum TC levels were significantly associated with adverse renal outcomes [21]. In addition, high-dose statins (>365 cumulative defined daily dose) significantly reduced the risks of all-cause mortality (hazard ratio (HR) 0.44, 95% CI 0.32–0.60), coronary artery disease (HR 0.20, 95% CI 0.13–0.31), cerebrovascular disease (HR 0.14, 95% CI 0.08–0.25), and end-stage renal disease (HR 0.22, 95% CI, 0.16 to 0.29) in SLE patients with hyperlipidemia [22]. In the current study, high levels of TC, TG, LDL-C, and VLDL-C were positively correlated with urea, creatinine, and uric acid, indicating that lipids were prone to cause kidney damage. In LN, the elevated urinary protein panel was detected in early stages of kidney injury [23]. We found that levels of TC, TG, LDL-C, and VLDL-C were positively correlated with 24 h M-TP, IgG, mAlb, TRU, A1M, and NAG of urine in LN children analyzed here. This also proved that dyslipidemia in children with SLE was closely related to the occurrence of kidney damage. The combined detection of TC, TG, LDL-C, and VLDL-C showed a sensitivity of 73.2% and a specificity of 79.0% for predicting LN in children. In addition, TC was positively and independently associated with the risk of LN (OR = 4.197, 95% CI: 1.133–15.545). The present results also showed that the relationship between the HDL-C level and LN was not clear, thus warranting further exploration.

The current study had a number of limitations. This was a retrospective single-center study, and thus, there may have been selection bias in the baseline characteristics. Further longitudinal studies and larger samples are therefore needed to explore underlying mechanisms.

## 5. Conclusion

Our study showed that dyslipidemia is common in children with LN and that the risk of LN is related to the blood lipid profile, which includes modifiable risk factors [24]. Dyslipidemia in children with lupus is often under-recognized and undertreated, and lipid profiles should thus be monitored regularly. Dyslipidemia should be managed aggressively, as early as possible, to prevent the deterioration of kidney function in these children. We aim to continue to assess the association between changes in lipid profiles and performance in LN over time and to analyze the effect of blood lipids on the prognosis of children with LN. The results of the current study lay the foundations for future studies.

## Figures and Tables

**Table 1:** Characteristics of the enrolled subjects.

Characteristics	LN group (*N* = 65)	Non-LN group (*N* = 69)	*T*, *χ*2 or *Z*	*P* value
Age, years	10.23 ± 2.57	10.64 ± 2.53	−0.923	0.358
Sex, no. (%)			0.414	0.520
Male	13 (20%)	17 (24.6%)		
Female	52 (80%)	52 (75.4%)		
BMI (kg/m^2^)	21.96 ± 7.91	20.73 ± 2.03	1.093	0.277
FBG (mmol/L)	5.13 ± 0.75	4.93 ± 0.77	1.569	0.119
ESR (mm/h)	36.31 ± 21.55	32.16 ± 25.17	0.993	0.323
C3 (g/L)	0.26 ± 0.14	0.55 ± 0.28	−7.615	0.001
C4 (g/L)	0.04 ± 0.03	0.10 ± 0.07	−6.154	0.001
Anti-dsDNA, no. (%)			4.705	0.030
Positive	50 (76.9%)	41 (59.4%)		
Negative	15 (23.1%)	28 (40.6%)		
Anti-Sm, no. (%)			3.350	0.067
Positive	21 (32.3%)	33 (47.8%)		
Negative	44 (67.7%)	36 (52.2%)		
SLEDAI score, no. (%)			−7.878	0.001
No activity (score: 0–4)	1 (1.5%)	30 (43.5%)		
Mild-to-moderate activity (score: 5–14)	31 (47.7%)	38 (55.1%)		
High activity (score: ≥ 15)	33 (50.8%)	1 (1.4%)		
Blood lipid profiles no. (%)			5.632	0.018
Normal blood lipids	7 (10.8%)	20 (29.0%)		
Dyslipidemia	58 (89.2%)	49 (71.0%)		

BMI: body mass index; FBG: fasting blood glucose; ESR: erythrocyte sedimentation rate; C3/C4: C3/C4 complement; anti-dsDNA: anti-double-stranded DNA antibody; anti-Sm: anti-Smith antibody; SLEDAI: systemic lupus erythematosus disease activity index.

**Table 2:** Blood lipid profile levels of the study groups.

Blood lipid profiles	LN group (*N* = 65)	Non-LN group (*N* = 69)	Healthy group (*N* = 70)	*P* value
TC ($\bar{x}$ ± *s*, mmol/L)	5.22 ± 1.88^ab^	4.20 ± 1.08^d^	3.94 ± 0.62	0.001
TG ($\bar{x}$ ± *s*, mmol/L)	2.27 ± 1.31^ab^	1.34 ± 0.59^c^	0.71 ± 0.41	0.001
HDL-C ($\bar{x}$ ± *s*, mmol/L)	1.10 ± 0.48^ae^	1.06 ± 0.44^c^	1.52 ± 0.31	0.541
LDL-C ($\bar{x}$ ± *s*, mmol/L)	3.05 ± 1.38^ab^	2.37 ± 0.84^c^	1.91 ± 0.52	0.001
VLDL-C ($\bar{x}$ ± *s*, mmol/L)	0.47 ± 0.27^ab^	0.27 ± 0.12^c^	0.14 ± 0.08	0.001
NEFA ($\bar{x}$ ± *s*, mmol/L)	0.45 ± 0.32^de^	0.52 ± 0.30^d^	0.54 ± 0.25	0.137

*Note.* a: compared with the healthy group, *P* < 0.05; b: compared with the non-LN group, *P* < 0.05; c: compared with the healthy group, *P* < 0.05; d: compared with the healthy group, *P* > 0.05; e: compared with the non-LN group, *P* > 0.05. Abbreviations: TC: total cholesterol; TG: triglyceride; HDL-C: high-density lipoprotein cholesterol; LDL-C: low-density lipoprotein cholesterol; VLDL-C: very low-density lipoprotein cholesterol; NEFA: nonesterified fatty acid.

**Table 3:** Relationships between blood lipid profiles and renal function indicators in children with LN.

Blood lipid profiles	24h M-TP	Urea	Cr	UA	IgG	mAlb	TRU	A1M	NAG
TC	0.412^a^	0.298^a^	0.249^a^	0.275^a^	0.347^a^	0.407^a^	0.280^a^	0.247^a^	0.157
TG	0.158	0.319^a^	0.196^a^	0.247^a^	0.217^a^	0.173	0.106	0.373^a^	0.270^a^
HDL-C	0.139	0.092	0.082	0.091	0.125	0.197	0.149	0.014	−0.067
LDL-C	0.408^a^	0.298^a^	0.247^a^	0.258^a^	0.361^a^	0.413^a^	0.293^a^	0.292^a^	0.192
VLDL-C	0.145	0.285^a^	0.173	0.283^a^	0.232^a^	0.195	0.121	0.397^a^	0.301^a^

*Note.* a: *P* < 0.05. Abbreviations: 24 h M-TP: 24-hour urine protein quantification; Cr: creatinine; UA: uric acid; IgG: urinary IgG; mAlb: urinary microalbumin; TRU: urinary transferrin; A1M: urinary *α*1 microglobulin; NAG: N-acetyl glucosidase.

## Data Availability

The data used to support the findings of this study are included within the article.
